# S10-1 Research on sub-national initiatives in HEPA policy making from Europe, Japan and the USA

**DOI:** 10.1093/eurpub/ckad133.048

**Published:** 2023-09-11

**Authors:** Chair(s):, Petru Sandu, Peter Gelius

**Affiliations:** Department of Public Health, University Babes-Bolyai; Department of Sport Science and Sport, Friedrich-Alexander-Universität Erlangen-Nürnberg

## Abstract

The importance of local governments' engagement in physical activity promotion at local level has been acknowledged and advocated by the World Health organization in documents such as: “Promoting physical activity and active living in urban environments: the role of local governments” or “Global action plan on physical activity 2018–2030: more active people for a healthier world”. However, given the relative “youngness” of the physical activity policy research domain, the local level initiatives have been underrepresented in the scientific literature. The current symposium aims to present a number of diverse initiatives to promote health enhancing physical activity at sub-national level, each having a very unique context and approach.

The paper from Eastern Michigan University presents the actions taken to implement Active People, Healthy Nation (APHN) program in geographic areas in Michigan where the priority population (Asian Americans) lives and the outcomes of this initiative.

The contribution from Japan describes the process of developing and applying the L-PAT (local policy audit tool), an instrument comprising 11 items based on WHO’s HEPA Policy Audit Tool (HEPA PAT) and the results of this survey within 47 Japanese prefectures and the C-PAT (city policy audit tool), a 6 items tool, in a selection of 272 Japanese municipalities.

The EU study presented in this symposium comprises the results of evaluation, using the CAPLA- Santé, a validated instrument, of local HEPA policies in 4 selected municipalities, one from each of the following countries: Finland, France, Germany, Romania and from one municipality in Japan.

The responsibilities of local authorities in health promotion and protection are undeniable. The way the authorities manage to fulfil these responsibilities is gaining more and more interest from various stakeholders from local level activists to researchers and policymakers at national and international level. Local communities are increasingly stimulated to contribute to their own wellbeing, so data on the status quo and progress is needed more than ever.

The initial discussion points of the symposium will focus on strengths and weaknesses of the initiatives presented, as well as the potential for knowledge exchange and replication & scaling-up.

